# Roles of neutrophil/lymphocyte ratio in prognosis and in differentiation of potential beneficiaries in HER2-positive breast cancer with trastuzumab therapy

**DOI:** 10.1186/s12885-020-06750-3

**Published:** 2020-03-19

**Authors:** Nianhua Ding, Juan Huang, Ningsha Li, Jiaqi Yuan, Shouman Wang, Zhi Xiao

**Affiliations:** 1 Department of clinical laboratory, The First Hospital of Changsha, Changsha, China; 2 Department of Breast Surgery, Xiangya Hospital, Central South University, 87 Xiangya Road, Changsha, Hunan People’s Republic of China 410008; 3 Clinical Research Center For Breast Cancer Control and Prevention In Human Province, Changsha, China

**Keywords:** Breast cancer, HER2-positive, Trastuzumab, Neutrophil/lymphocyte ratio (NLR)

## Abstract

**Background:**

The relationship of neutrophil/lymphocyte ratio (NLR) to prognosis of HER2-positive breast cancer (BC) is not well studied. We aimed to assess the prognostic role of NLR in HER2-positive BC patients treated with or without trastuzumab.

**Methods:**

The clinical data of 843 HER2-positive BC patients from July 2013 to July 2018 were collected. The difference among variables was calculated by chi-square test. The associations between clinicopathological factors, NLR and disease-free survival (DFS) were analyzed by univariate and multivariate analyses.

**Results:**

Patients were divided into three groups. In group 1 containing 255 patients without trastuzumab treatment, pretreatment NLR showed no predictive value. Patients with trastuzumab treatment were divided into two groups on equal, according to pretreatment NLR values, low NLR (group 2) and high NLR (group 3). Patients in group 2 showed significantly higher 3-year DFS rate than patients in group 1 and group 3 (95.3% vs. 91.6% vs. 90.5%, respectively, *P* = 0.011); patients in the group 1 and group 3 had a similar 3-year DFS outcome. Multivariate analysis showed high pretreatment NLR was significantly associated with shorter DFS (HR = 2.917, 95% CI = 1.055–8.062, *P* = 0.039) in HER2-positive BC patients treated with trastuzumab.

**Conclusions:**

Among HER2-positive trastuzumab-treated BC patients, low pretreatment NLR value was associated with better DFS, and it might help to differentiate potential beneficiaries of trastuzumab treatment.

## Background

HER2, which is associated with aggressive tumor growth and poor prognosis, is over-expressed in 20–25% of all invasive breast cancer (BC). Trastuzumab (Herceptin®) is an effective therapy for HER2 over-expression BC and can decrease the risk of relapse by around 25% in the adjuvant setting [1]. The anti-tumor mechanisms of trastuzumab might be to prevent ligand binding and dimer formation, to inhibit kinase of downstream signaling partners, to induce antibody-dependent cell-mediated cytotoxicity (ADCC), and so on [2]. Meanwhile, the situation that HER2-positive BC has no response to trastuzumab treatment exists indeed [3]. The mechanism of trastuzumab resistance is not yet understood, and there is no factor that could predict the sensitivity to trastuzumab of HER2-positive breast cancer [4, 5].

Recently, many studies have found that inflammation and immunity play critical roles in tumor initiation, invasion, and metastasis [6]. Host anti-tumor immunity might offer a new strategy for curing BC [7–9]. The basic immune surveillance of the body and changes in systemic immune status, affect responses to therapy and even prognosis of BC patients. Nevertheless, there are no authorized biomarkers reflecting systemic immune status [8]. Some studies indicated that low pretreatment neutrophil/lymphocyte ratio (NLR) was predictive factor of better DFS outcome in BC, especially in triple-negative BC patients [10–12]. In the HER2-positive subtype, few studies have explored the relationship between NLR and DFS of patients with or without trastuzumab treatment. Moreover, two recent studies showed the different results concerning the relationship between absolute lymphocyte account and disease progression in HER2-positive BC [13, 14]. In this study, we retrospectively analyzed the NLR as the predictive factor of 843 HER2-positive BC patients with or without trastuzumab treatment.

## Methods

### Patients background

A total of 843 female patients with HER2-positive invasive BC were successively collected in the Breast Department of Xiangya Hospital of Central South University from July 2013 to July 2018. Among them, 588 patients had received trastuzumab treatment for 1 year, and 255 patients had not. Medical records were reviewed to collect and organize on patients’ age, medical history, laboratory test, and pathologic results such as tumor size, histological grade, lymph node status, and hormone receptor status. Patients were treated with neoadjuvant chemotherapy (NC) or adjuvant chemotherapy mainly including anthracycline and/or taxane regimens. All these patients had undergone surgery including breast-conserving or mastectomy and sentinel lymph node biopsy or modified radical mastectomy. Post-surgical radiotherapy and endocrine therapy were recommended according to normal practice. We excluded patients with inflammatory breast cancer, multiple tumors, acute and chronic injury, acute and chronic inflammation, hematological disorders, liver cirrhosis, and end-stage renal disease. This study was approved by the Institutional Review Board of Xiangya Hospital.

### Pathological characteristics

Hormone receptor (HR) was considered positive if ER or PR were positive. ER and PR were evaluated as positive if there was at least 1% positive invasive tumor nuclei in the samples. HER-2 positive was defined as immunohistochemistry staining of 3+, immunohistochemistry staining of 2+ but FISH positive as HER-2/CEP17 ratio > 2.2.

### Laboratory data

The NLR was defined as the absolute neutrophil count (N) divided by the absolute lymphocyte count (L). The pretreatment NLR was defined as pre-NLR and calculated from the full blood count routinely performed within 1 week before any treatment. There were no signs of clinical infection such as fever on the day of blood collection.

### Grouping

Eight hundred forty-three patients were divided into three groups. Group 1 included 255 patients without trastuzumab treatment. The reason of no trastuzumab treatment was that Trastuzumab (Herceptin®) was not covered by insurance until July of 2016 and these patients could not afford it. Five hundred eighty-eight patients treated with trastuzumab were divided into two groups on average, group 2 and group 3, according to the values of pre-NLR. The cutoff value of NLR was 1.830. Group 2 included 294 patients with low pre-NLR value and group 3 included 294 patients with high pre-NLR value.

### Statistical analysis

Pearson χ2 test was performed to compare categorical parameters of clinicopathological characteristics. For survival analysis, the endpoint was DFS. DFS was defined as the length of time to survive without any signs or symptoms of relapse or metastasis after surgery. Kaplan-Meier curves and log-rank tests were performed to assess differences among groups. Multivariate Cox regression models were established to identify significant predictors of DFS. Statistical significance was calculated at the 95% confidence interval (*P* < 0.05), and all analyses were carried out using SPSS version 17.0 for Windows (SPSS Inc., Chicago, USA) as previously [15].

## Results

### Clinicopathological characteristics of 843 patients with HER2-positive BC

Eight hundred forty-three patients were divided into three groups as mentioned above. Detailed distributions of patients with different age, tumor size, lymph node status, and histological grade were summarized in Table 1. The median follow-up was 20 months (6 to 50 months). Among the three groups, there were more women less than or equal to 40 years old and with tumor of large size in group 2, when comparing with that in group 1 or group 3. There were no significant differences in lymph node status, histological grade, hormone status, Ki67 score or NC status among these three groups (*P* > 0.05).

**Table 1:** Comparison of clinicopathological characteristics of 843 HER2-positive patients

Factors	Without trastuzumab	With trastuzumab		*p*-value
	Group 1 n(%)	Group 2 n(%)	Group 3 n(%)	
Age (year)				
≤ 40	31 (12.2)	76 (25.9)	44 (15.0)	**< 0.001**
> 40	224 (87.8)	218 (74.1)	250 (85.0)	
T stage				
T1	51 (20.0)	70 (23.8)	64 (21.8)	**0.044**
T2	172 (67.5)	172 (58.5)	200 (68.0)	
T3–4	32 (12.5)	52 (17.7)	30 (10.2)	
N stage				
pN0	144 (56.5)	162 (55.1)	152 (51.7)	0.217
pN1	76 (29.8)	78 (26.5)	79 (26.9)	
pN2–3	35 (13.7)	54 (18.4)	63 (21.4)	
Histological grade				
1~2	198 (77.6)	246 (83.7)	242 (82.3)	0.171
3	57 (22.4)	48 (16.3)	52 (17.7)	
HR				
Negative	145 (56.9)	164 (55.8)	164 (55.8)	0.957
Positive	110 (43.1)	130 (44.2)	130 (44.2)	
Ki67 (%)				
< 30	67 (52.8)	134 (45.6)	132 (44.9)	0.187
≥ 30	60 (47.2)	160 (54.4)	162 (55.1)	
NC				
No	117 (45.9)	128 (43.5)	130 (44.2)	0.853
Yes	138 (54.1)	166 (56.5)	164 (55.8)	
Total	255 (100.0)	294 (100.0)	294 (100.0)	

Group 1 contained patients without trastuzuamb treatment, group 2 contained patients with trastuzuamb and low pre-NLR value, and group 3 contained patients with trastuzuamb and high pre-NLR value. Statistically significant factors are in bold font. NLR, neutrophil/lymphocyte ratio; HR, hormone receptor; NC, neoadjuvant chemotherapy

The treatment details for patients were summarized in Table 2. Among them, 689 patients received epirubicin and cyclophosphamide followed by the taxane regimen. Four hundred sixty-four patients received radiation therapy and 379 patients received endotherapy. There was no difference among the three groups.

**Table 2:** Treatment for 843 HER2-positive BC patients

Treatment	Without trastuzumab	With trastuzumab		*p*-value
	Group 1 n(%)	Group 2 n(%)	Group 3 n(%)	
Chemotherapy regimen				
EC-T	203 (79.6)	248 (84.4)	238 (81.0)	0.846
TCb	37 (14.5)	35 (11.9)	39 (13.3)	
P	8 (3.1)	6 (2.0)	10 (3.4)	
Others	7 (2.7)	5 (1.7)	7 (2.9)	
Radiotherapy				
No	114 (44.7)	124 (42.2)	141 (48.0)	0.369
Yes	141 (55.3)	170 (57.8)	153 (52.0)	
Endotherapy				
No	142 (55.7)	162 (55.1)	160 (54.4)	0.956
Yes	113 (44.3)	132 (44.9)	134 (45.6)	
Total	255 (100.0)	294 (100.0)	294 (100.0)	

Group 1 contained patients without trastuzuamb treatment, group 2 contained patients with trastuzuamb and low pre-NLR value, and group 3 contained patients with trastuzuamb and high pre-NLR value. EC-T**,** epirubicin and cyclophosphamide followed by the taxane; TCb, docetaxel and carboplatin; P, taxane; NLR, neutrophil/lymphocyte ratio

### DFS outcome among patients with trastuzumab treatment (group 2 and 3)

Twenty six events occurred without local recurrence among 588 patients during the follow-up. There were eight patients with multiple metastatic sites, eight patients with metastases in lung, six patients with metastases in brain, and four patients with metastases in bone or contralateral supraclavicular lymph nodes.

**Fig. 1:**
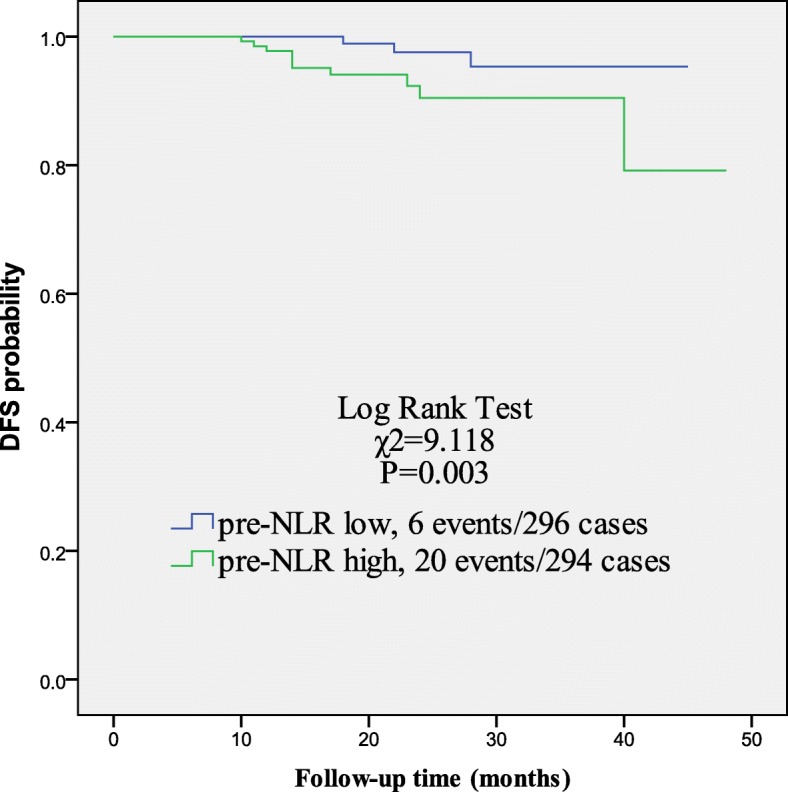
Kaplan-Meier curves for the DFS outcomes among patients with trastuzumab. Patients with low pre-NLR values (group 2) showed higher 3-year DFS compared with that with high pre-NLR values (group 3) (*p* = 0.003). DFS, disease-free survival; pre-NLR, pretreatment neutrophil/lymphocyte ratio

Kaplan-Meier (KM) curve was used to analyze the prognostic factor. As shown in Fig. 1, patients in group 3 showed significantly lower 3-year DFS rate than patients in group 2 (90.5% vs. 95.3%, *P* = 0.003).

As shown in Table 3, patients less than or equal to 40 years old, with higher histological grade or more lymph nodes involved, with HR negative tumor had a significantly worse DFS outcome than patients older than 40, with lower histological grade or less lymph nodes involved, with HR positive tumor. Cox regression model was used for the evaluation of DFS rate among patients treated with trastuzumab. Table 3 showed that younger age, more lymph nodes involved, HR negative and high pre-NLR values were independent prognostic factors of worse outcome for the HER2-positive patients with trastuzumab treatment. The risk of metastasis was higher in group 3 compared to that in group 2 (HR = 2.917, 95% CI = 1.055–8.062, *P* = 0.039).

**Table 3:** Univariate and multivariate analyses for DFS among patients with trastuzumab

Factor	Univariate	Multivariate		*p*-value
	*p*-value	HR	95%CI	
Age (≤40 vs. > 40)	**< 0.001**	3.907	1.551–9.840	**0.004**
T stage	0.417			
(T2 vs. T1) (T3 vs. T1)				
pN stage	**< 0.001**			
(pN1 vs. pN0)		2.930	0.832–10.320	0.094
(pN2–3 vs. pN0)		9.478	3.026–29.681	**< 0.001**
Grade (3 vs. 1–2)	**0.013**			0.435
HR (negative vs. positive)	**0.001**	5.854	1.878–18.245	**0.002**
Ki67 (≥30% vs. < 30%)	0.574			
pre-NLR (high vs. low)	**0.003**	2.917	1.055–8.062	**0.039**
NC (yes vs. no)	0.333			

HR, hazard ratio; CI, confidence interval; NC, neoadjuvant chemotherapy; NLR, neutrophil/lymphocyte ratio; ALC, absolute lymphocyte count. Statistically significant factors are in bold font

### DFS outcome among patients without trastuzumab treatment (group 1)

**Fig. 2:**
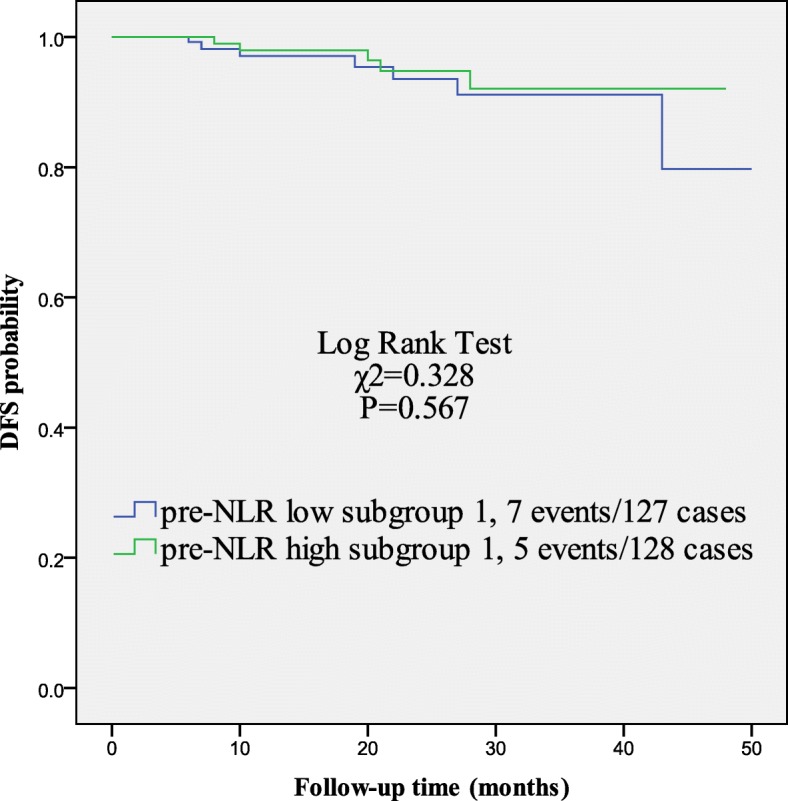
Kaplan-Meier curves for the DFS outcomes among patients without trastuzumab (group 1). Patients in group 1 (255) were divided into two subgroups on average, low and high pre-NLR subgroup 1, according to the values of pre-NLR. Patients in low and high pre-NLR subgroup 1 showed no significant difference in DFS outcome. DFS, disease-free survival; pre-NLR, pretreatment neutrophil/lymphocyte ratio

Twelve metastatic events occurred without local recurrence in group 1 (255 patients) during the follow-up. There were two patients with bone metastatic lesion and one patient with liver lesion. The rest of nine patients all had multiple metastases. We assessed whether pre-NLR value could be a prognostic factor in HER2-positive patients without trastuzumab treatment. Patients in group 1 (255) were divided into two subgroups on average, low and high pre-NLR subgroup 1, according to the values of pre-NLR. As shown in Table 4 and Fig. 2, there were no significant differences in clinicopathological factors status and DFS outcome between low and high pre-NLR subgroups.

**Table 4:** Comparison of clinicopathological characteristics of 255 HER2-positive patients without trastuzumab

Factors	Group 1		*p*-value
	Low pre-NLR	High pre-NLR	
Age (year)			
≤ 40	14 (11.0)	17 (13.3)	0.581
> 40	113 (89.0)	111 (86.7)	
T stage			
T1	29 (22.8)	22 (17.2)	
T2	82 (64.6)	90 (70.3)	0.515
T3–4	16 (12.6)	16 (12.5)	
N stage			
pN0	68 (53.5)	76 (59.4)	
pN1	41 (32.3)	35 (27.3)	0.624
pN2–3	18 (14.2)	17 (13.3)	
Histological grade			
1~2	98 (77.2)	100 (78.1)	0.854
3	29 (22.8)	28 (21.9)	
HR			
Negative	72 (56.7)	73 (57.0)	0.957
Positive	55 (43.3)	130 (43.0)	
Ki67 (%)			
< 30	67 (52.8)	57 (44.5)	0.189
≥ 30	60 (47.2)	71 (55.5)	
NC			
No	59 (46.5)	58 (45.3)	0.855
Yes	68 (53.5)	70 (54.7)	
Total	127 (100.0)	128 (100.0)	

*NLR* neutrophil/lymphocyte ratio, *HR* hormone receptor, *NC* neoadjuvant chemotherapy

### DFS outcome among three groups of 843 HER2-positive BC patients

**Fig. 3:**
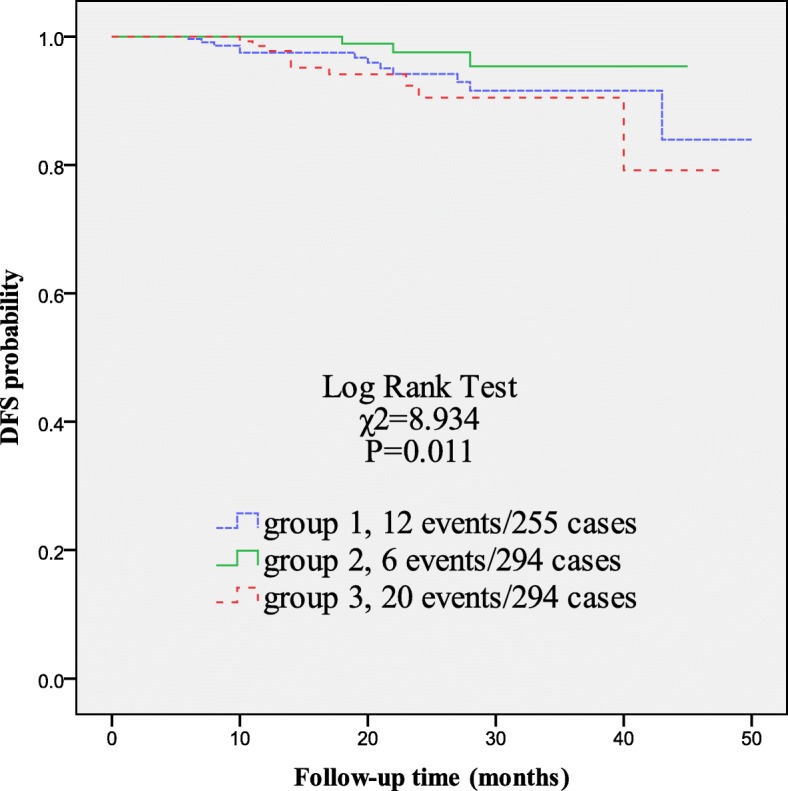
DFS outcome among three groups of 843 HER2-positive BC patients. Patients in group 2 showed significantly higher 3-year DFS rate than patients in group 1 or group 3. Patients in the group 1 and group 3 had similar 3-year DFS outcome. Group 1 (without trastuzumab treatment), group 2 (with trastuzumab treatment and low pre-NLR value) and group 3 (with trastuzumab treatment and high pre-NLR value)

As mentioned above, 843 HER2-positive BC patients had been divided into three groups: group 1 (without trastuzumab treatment), group 2 (with trastuzumab treatment and low pre-NLR value) and group 3 (with trastuzumab treatment and high pre-NLR value). KM curves were used to analyze the DFS outcomes among the three groups. As shown in Fig. 3, patients in group 2 showed significantly higher 3-year DFS rate than patients in group 1 and group 3 (95.3% vs. 91.6% vs. 90.5%, respectively, *P* = 0.011); patients in the group 1 and group 3 had similar 3-year DFS outcome.

## Discussion

In this study, we analyzed the effect of some conventional prognostic factors such as age, tumor size, nodal involvement, tumor grade, hormone status, and the inflammatory predictor, NLR, on the DFS outcome among HER2-positive patients with or without trastuzumab therapy. Patients older than 40 years, with fewer nodes involved and HR-positive tumor were associated with favorable DFS outcome in HER2-positive BC patients receiving trastuzumab treatment. And the pretreatment NLR was identified to be an independent predictive factor among trastuzumab-treated patients. However, pretreatment NLR showed no predictive value among HER2-positive patients without trastuzumab treatment. More information will be needed to validate whether pretreatment NLR could help us to distinguish patients with HER2-positive BC who will benefit from trastuzumab treatment or not.

NLR is a routinely available marker of the systemic inflammatory response, and there is no significant difference of NLR value in distinct breast cancer subtype [16]. The presence of higher NLR in the blood has been recognized as a poor prognostic factor among triple-negative BC patients [10, 11]. Meanwhile, a meta-analysis suggested that NLR was a good prognostic marker for HER2-positive BC and triple-negative BC, but not for luminal A and luminal B subtype BC [17]. However, there were not sufficiently addressed about trastuzumab use for the HER2-positive BC patients in the meta-analysis. Another retrospective study of 187 HER2-positive BC patients receiving adjuvant trastuzumab implied that low pretreatment NLR might be associated with improved DFS outcome, but without significant difference [18]. In this study, first we categorized the HER2-positive BC patients according to whether they had received trastuzumab therapy or not. Data of patients without trastuzumab verified there were no predictive value of pretreatment NLR, but data about trastuzumab-treated patients showed low pretreatment NLR values were associated with improved survival. The reason was not yet well understood. Neutrophils are recognized as not only important contributors to tumor progression, metastasis and production of proangiogenic factors, but also inhibitors of activity of T cells and natural killer cells through production of arginase-1 and hydrogen peroxide [19–23]. Lymphocytes are important factors of immune surveillance and immune response, especially in the tumor microenviroment where tumor-infiltrating lymphocytes might be associated with chemotherapy response and survival outcomes [24]. In the HER2-positive BC treated with trastuzumab, trastuzumab-induced ADCC should be taken into consideration for its contribution to the improved DFS outcome when compared with those without trastuzumab treatment [25]. The intensity of ADCC induced by trastuzumab might be different for various reasons, such as HER2 copy numbers/application, FcγIIIA/FcγIIA polymorphisms, and so on [26–28]. However, there is no study about the correlation of trastuzumab response and host immune status.

Then we divided patients into three groups for analysis of DFS outcome. As shown in Table 1 and Fig. 3, patients in group 1 had similar clinicopathological characteristics and 3-year DFS outcome as that in group 3. However, patients in group 3 were treated with trastuzumab which was supposed to decrease the risk of metastasis around 25% in the adjuvant setting comparing to patients without trastuzumab [1]. Meanwhile, patients in group 2 showed significantly higher 3-year DFS rate than patients in group 1 and group 3 (Fig. 3). This implied that HER2-positive BC patients with high pretreatment NLR values might not benefit from trastuzumab treatment or might benefit very little, and HER2-positive patients with low pretreatment NLR value would benefit from trastuzuamb. This might be explained by the trastuzumab-induced ADCC, one of anti-tumor mechanisms of trastuzumab, whose ability might be associated with host immune status. Trastuzumab might induce the ADCC function against metastasis of breast cancer more efficiently, if the host immune status is in good condition (low NLR status), and trastuzumab might not induce the ADCC function if host immune system was in suppressive condition (high NLR status). In brief, we deduced that trastuzumab could bring out stronger anti-tumor immune competence through ADCC function in patients with low pretreatment NLR value than those with high pretreatment NLR value. More evidence and researches are needed for the exploration of trastuzumab’s anti-tumor efficacy under different immune status. The reanalysis of some classical trials, such as HERceptin Adjuvant (HERA) trial, NSABP B-31 and NCCTG 9831, might provide more useful information about NLR in predicting prognosis of HER2-positive BC.

In this study, we also analyzed posttreatment NLR which were obtained 1 month after the chemotherapy or radiotherapy, and no significant association was found between NLR and DFS outcome in trastuzumab-treated patients (data not shown). In our opinion, the pretreatment NLR which was obtained within 1 week before any treatment was more representative of baseline immune status than posttreatment NLR which might be affected by chemotherapy and/or radiotherapy, even though NLR was calculated from blood data 1 month after last chemotherapy or radiotherapy. This was in line with most of studies that showed the low baseline NLR was associated with better survival outcomes [29–32].

There are some limitations in this study. First, this study is a retrospective analysis of HER2-positive patients only in one hospital. Data were uneven among group1, group 2 and group 3. In the group 2, more patients were less than or equal to 40 years old and with large tumor size. Tumor size was not a prognostic factor in this study. Nevertheless age was an independent predictor and younger age was associated with worse survival outcome. However this did not affect the results from multivariate analysis which showed patients in group 2, which had more younger BC patients and was supposed to have a worse prognosis, actually had better 3-year DFS rate. Second, the median follow-up was only 20 months and only 38 events occurred among all these patients. It was not long enough to obtain more events, and longer follow-up time is needed. Third, the time point of trastuzumab therapy was not exactly the same among patients. Some of patients had received trastuzumab during neoadjuvant therapy, and some were treated after operation. Fourth, we did not analyze the tumor infiltrating lymphocytes (TILs) in tumor microenvironment which were well recognized parameter as prognostic factor for HER-2 positive BC. More analysis are needed for validating the correlation between NLR and TILs. Re-analyses of classical clinical trials are needed for the exploration of the relationship between systemic inflammatory/immune markers and trastuzumab-induced ADCC function.

## Conclusions

This study showed that low pretreatment NLR was a predictive factor of better DFS outcome among HER2-positive BC receiving trastuzumab therapy. Pretreatment NLR value might help to distinguish HER2-positive BC patients who will benefit from trastuzumab treatment from those who will not. Re-analyses of classical clinical trials are needed to verify the role of pretreatment NLR in HER2-positive BC treated with trastuzumab.

## Data Availability

The datasets generated and/or analysed during the current study are not publicly available due to its usage for another article, but are available from the corresponding author on reasonable request.

## Abbreviations

NLR: Neutrophil/lymphocyte ratio
BC: Breast cancer
DFS: Disease-free survival
ADCC: Antibody-dependent cell-mediated cytotoxicity
NC: Neoadjuvant chemotherapy
HR: Hormone receptor
KM: Kaplan-Meier
